# Categorization of differences of sex development among Egyptian children and the role of antimullerian hormone and inhibin B

**DOI:** 10.3389/fendo.2022.1072399

**Published:** 2023-01-06

**Authors:** Shereen Abdelghaffar, Engy Nasr AbdelMoneam, Samah A. Hassanein, Noha Abdelhalim Radwan, Marwa Farouk Mira

**Affiliations:** ^1^ The Diabetes, Endocrine and Metabolism Pediatric Unit, Pediatric Department (DEMPU), Cairo University, Cairo, Egypt; ^2^ Department of Clinical and Chemical Pathology, Cairo University, Cairo, Egypt

**Keywords:** anti-mullerian hormone, inhibin B, congenital adrenal hyperplasia, androgen insensitivity syndromes, atypical genitalia, differences of sex development

## Abstract

**Background:**

Differences of sex development (DSD) are congenital conditions linked to atypical development of chromosomal, gonadal, or anatomical sex.

**Objective:**

The aim of this study was to demonstrate our experiences at the Diabetes Endocrine and Metabolism Pediatric Unit (DEMPU), Faculty of Medicine, Cairo University in the field of DSD by focusing on the clinical presentation, laboratory profile, classification, and etiological diagnosis of these conditions. In addition, the present study intended to delineate the importance of serum anti-Müllerian hormone (AMH) and inhibin B in detecting the presence of functioning testicular tissue.

**Methods:**

This cohort study included 451 infants and children with various clinical presentations of DSD. The study performed a retrospective analysis on medical records of established DSD cases to evaluate the clinical importance of AMH and inhibin B. In addition, newly diagnosed patients were prospectively analyzed.

**Results:**

Three hundred thirty-six (74.5%) patients were 46,XY DSD, 98 (21.7%) were 46,XX DSD, 14 patients had other karyotypes and 3 had missing karyotypes. Among the 46XY DSD patients, the most common cause was partial androgen insensitivity. In contrast, congenital adrenal hyperplasia constituted the most common diagnosis in 46,XX DSD cases. The cut off value of serum AMH was 14.5 ng/ml with 100% sensitivity and 55.1% specificity.

**Conclusion:**

Partial androgen insensitivity was the most important cause of 46,XY DSD in Egyptian children, and congenital adrenal hyperplasia was the most common cause of 46,XX DSD. AMH was valuable in detecting functioning testicular tissue.

## 1 Introduction

Differences of sex development (DSD) area group of congenital conditions linked to atypical development of chromosomal, gonadal, or anatomical sex (1). DSD is associated with many clinical presentations, the most common of which is atypical genitalia (2). The global incidence of DSD is nearly 1 in 4500 to 5500 (3), while its incidence in Egypt has been estimated to be 1 in 3,000 live births (4). The most common cause of atypical genitalia is congenital adrenal hyperplasia (2), followed by androgen insensitivity (5).

Meticulous history, detailed physical examination, and biochemical investigations, including karyotyping, radiological, and genetic testing, are important diagnostic processes to determine the etiology of DSD, subsequently allowing the implementation of appropriate management strategies, such as hormonal replacement and gonadal malignancy risk stratification (6, 7).

In children with 46,XY DSD, serum anti-Müllerian hormone (AMH) and/or inhibin B, as well as basal or post-human chorionic gonadotropin (HCG)stimulation test androgens including, androstenedione, testosterone, and dihydrotestosterone levels (DHT), are necessary in order to assess androgen synthesis and mechanism of action (8). In patients with impalpable gonads, serum AMH and inhibin B can also detect the presence of functioning testicular tissue secreted by sertoli cells (9). The aim of the present study was to demonstrate our experiences at the Diabetes Endocrine and Metabolism Pediatric Unit (DEMPU), Faculty of Medicine, Cairo University in the field of DSD in light of the increased number of referrals of children presenting with atypical genitalia to our tertiary care hospital from across Egypt. In addition, we focused on determining the etiological distribution and clinical and laboratory profile among DSD patients, and evaluate the importance of AMH and inhibin B in detecting functioning testicular tissue.

## 2 Materials and methods

This was a longitudinal cohort study, which included 451 infants and children with DSD, followed at the DEMPU of Cairo University Children's Hospital between November2019 and January 2022.The minimum age was seven days, and the maximum age was 14 years. The present study was approved by the Cairo University Hospitals Research Committee on 12-10-2019 (MD-92-2019). Records of infants and children who had already been diagnosed with DSD were retrospectively analyzed, while new cases presented to our clinical settings during the timeline of the study were prospectively analyzed.

All patients were subjected to history taking, including atypical genitalia, salt-losing crisis, consanguinity, and family history of infertility.

*Physical examination*: A comprehensive physical examination was performed in a private setting after explaining the examination steps and obtaining informed consent from the children’s parents or caregivers.

*General examination*: Participants’ blood pressure and other vitals that would indicate signs of dehydration were measured. Furthermore, we evaluated potential dysmorphic features or any other associated anomalies, and we used Tanner staging for pubertal assessement (10).

*Genital examination*: Different degrees of virilization in female participants were assessed with the Prader scale (11). In addition to calculating the external masculinization score (EMS), the Quigley scale was used to assess the development of external genitalia in 46,XY children (12, 13).

The following investigations were also performed:

1. Abdomino-pelvic scrotal ultrasonography (U/S) was performed as the first-line screening imaging modality.
2. Laboratory investigations: All blood samples were collected under complete aseptic conditions; *karyotype analysis* (peripheral blood): 3–5 mL in sodium/ lithium heparin vacutainer tube. *Hormonal evaluation*: 17-Hydroxyprogesterone was used to screen for CAH secondary to 21-hydroxylase deficiency; Dehydroepiandrosterone (DHEA), progesterone, and 11-deoxycortisol were used to diagnose less common forms of CAH; Testosterone, its precursors and metabolites (DHT and Δ4 androstendione), follicle-stimulating hormone, and luteinizing hormone were evaluated in patients with 46,XY DSD. Basal cortisol and adrenocorticotropic hormone (ACTH) were measured to determine the diagnosis of panhypopituitarism and enzymatic disorders affecting adrenal steroidogenesis. Human chorionic gonadotrophin (HCG) stimulation tests, including measurements of basal testosterone, DHT, and Δ4 androstenedionelevels, were performed, and then 1000–1500 IU (<1 year old, 500 units; 1–10 years, 1000 units; >10 years, 1500 units) of HCG were given intramuscularly (IM) daily for three consecutive days. Consequently, potential increase in testosterone, DHT, and Δ-4 androstenedione levels were assessed 24 hours after the last HCG injection. In cases where testosterone response to the standard 3-day HCG stimulation test was poor, long HCG stimulation was subsequently indicated. Administration of 1500 IU HCG twice weekly for the following 2 weeks was then performed, with a total of seven injections in three weeks and samples were withdrawn on day 22 to assess the testosterone function (14, 15). Response to HCG was considered normal when the absolute testosterone concentrations reached a higher value than the upper limit of the normal prepubertal range or increased by more than twofold the baseline value (16). Increased testosterone levels after HCG testing is suggestive of 5-alpha-reductase deficiency or androgen insensitivity. Stimulated T: DHT ratio>30 is likely to be 5-alpha-reductase-2 deficiency (17, 18). The different etiologies of DSD were diagnosed according to the guidelines of the British Society for Endocrinology (18). Serum AMH levels and previous hormonal profiles were collected from patients’ files.

In this study, we assessed the serum inhibin B levels of patients with 46,XY DSD using enzyme-linked immunoassay (ELISA).The normal reference range for AMH and inhibin B were as follows (19) :Inhibin B (ng/dl): <one month (133–196); one month < one year (195–256); 1–10 years (91–163); and 10–20 years (169–216). AMH (ng/ml): <14 days (35–140); 15 days– six months (55–210); six months– two years (85–320); 2–9 years (55–250); 9–18 years: Tanner 1(35–200), Tanner 2(10–140), Tanner 3 (4–55), Tanner 4 (4–22), and Tanner 4 (4–21).

3. Interventional investigations: a) In fluoroscopy-Genitography, genitograms were performed in preparation for surgery in 46,XX virilized females.b)Laparoscopic procedures were performed in 46,XX DSD patients to assess the urogenital anatomy. Contrary to patients with 46,XY DSD, endoscopic examination was performed for the identification of any Mullerian remnants and gonads in all patients with impalpable testes.

### 2.1 Statistical analysis

Data were statistically described as mean ± standard deviation (SD), median and range or frequencies (number of cases), and percentages when appropriate. The assumption of normality for numerical data was tested using the Kolmogorov Smirnov test. Comparisons between the study groups were performed using Student *t* test for independent samples when comparing two groups of normally distributed data and/or large enough samples, and using the Mann–Whitney *U* test for independent samples when comparing not-normal data. Also, we used the Kruskal–Wallis test to compare normally distributed numerical variables among groups, and the Chi-square (χ^2^) test was used to compare categorical data. However, the exact test was employed when the expected frequency was less than five. Spearman rank correlation equation was used to demonstrate correlations among various variables. Two-sided *p* values less than 0.05 were considered statistically significant. The IBM SPSS (Statistical Package for the Social Science; IBM Corp, Armonk, NY, USA) release 22 for Microsoft Windows was used for all statistical analyses. The cut off value of serum AMH was detected using ROC curve.

## 3 Results

**Figure 1:**
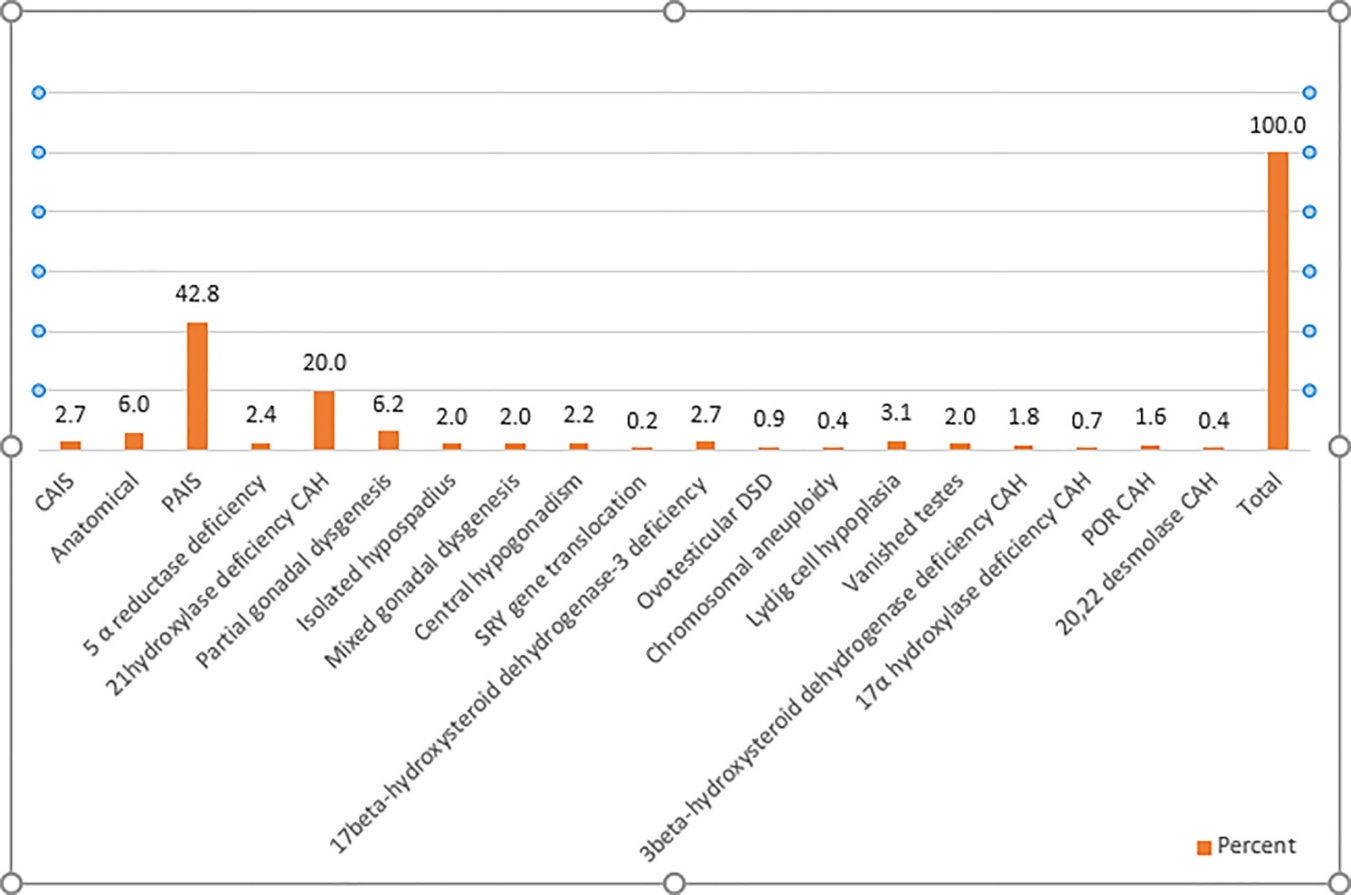
Etiological classification of cases of DSD. CAH, congenital adrenal hyperplasia; CAIS, complete androgen insensitivity; DSD, differences of sex development; PAIS, partial androgen insensitivity; POR CAH, P450 oxidoreductase deficiency.

This study included 451 infants and children with DSD of which 336 patients (74.5%) had 46,XY DSD, 98 patients (21.7%) had 46XX DSD, and only 14 patients (3.1%) had another karyotype, including 10 patients with mixed gonadal dysgenesis, one with Klinefelter syndrome (47 XXY), one with trisomy 21/ Klinefelter syndrome (47XXY/ 48 XXY), one patient with trisomy 18, and one with trisomy 13. There were three patients for which karyotype results could not be obtained. **Figure 1** summarizes the spectrum of different etiologies of DSD among the patients included in this study. PAIS was the most prevalent diagnosis, representing 42.8 % of all patients, while CAH represented 24.2 % of the population study, with 21 hydroxylase deficiency being the most common form of CAH.

In addition to one patient with Robinow syndrome and two patients with cloacal dystrophy and associated renal anomalies, respectively, the associated anomalies primarily included the characteristic features of trisomy 21, 13, and 18. Other disorders included congenital heart defects, limb anomalies, and dysmorphic features. Moreover, one patient had a double uterus, double vagina, and imperforate anus.

The mean age of presentation among non salt-losing individuals was 3.45 years (± SD 3.865). **Table 1** outlines the clinical, imaging, and laparoscopic findings of the gonads of patients with DSD.

**Table 1:** Clinical criteria, imaging, and laparoscopic findings of patients with DSD.

	Number (N)	Percent %
• Number of genital openings		
Single opening	383	84.9
Vaginal and urethral openings	68	15.1
• Site of the single genital opening		
Normal	109	28.1
Penile	35	9.0
Penoscrotal	240	61.9
Perineal	4	1.0
• Site of the right gonad (clinical examination)		
Scrotal		50.14
Inguinal		30.14
Nonpalpable		19.72
• Site of the left gonad (clinical examination)		
Scrotal		53.4
Inguinal		25.4
Nonpalpable		21.2
• Imaging of the right gonad		
Scrotal	188	53
Inguinal	140	39.4
Nonvisualized	27	7.6
• Imaging of the left gonad		
Scrotal	178	50.1
Inguinal	147	41.4
Nonvisualized	30	8.5
• Laparoscopy of the right gonad		
Present	17	70.8
Absent	7	29.2
Dysgenetic	2	12.5
Testis	8	50
Ovary	5	31.2
Ovotestis	1	6.3
• Laparoscopy of the left gonad		
Present	14	58.3
Absent	10	41.7
Dysgenetic	2	14.3
Testis	7	50
Ovotestis	3	21.4
Ovary	2	14.3
• Prader scoring of viralized females (Total score: 5)		
1		3.8%
2		23.8%
3		38.1%
4		29.5%
5		4.8%
• Quigley score for underviralized males (Total score: 7)		
1		8.0%
2		15.1%
3		46.3%
4		14.8%
5		8.8%
6		7.1%
7		0.0%
• External masculinizing score (total score: 12)		
1	5	1.4
2	59	16.6
3	107	30.4
4	4	1.1
5	36	10.2
6	49	13.9
7	5	1.4
8	30	8.5
9	26	7.4
10	4	1.1
11	19	5.4

DSD, Differences of sex development.

Our results showed a significant correlation between the Quigley score and initial sex of rearing (*p*<0.001). Compared to 51.6% of 46,XY DSD patients with a Quigley score of 5, all 46,XY DSD patients with a Quigley score of 1–2 were initially reared as males. Among virilized females, all patients with a Prader score of 1 were initially reared as females. In contrast, 60% of patients with a Prader score of 5 were initially reared as males (**Table 2**). **Table 2** also demonstrates the initial sex of rearing for patients with different etiological diagnoses. In patients with 46,XY DSD, Comparison between hormonal profile of patients with poor HCG response and those with normal HCG response was outlined in **Table 3**.The minimum of post HCG stimulation serum total testosterone level in patients with good response in this study was 0.2 ng/ml, which was near the normal prepubertal level and > twofold the basal testosterone level for this patient (0.02ng/ml).

**Table 2:** Relationship between initial sex of rearing and different etiologies and scores of DSD.

	Initial Rearing	N (%)	*P* Value
	N (%)		
• Etiology	Female	Male	<0.001
CAIS	12 (100%)	0 (0%)	
PAIS	43 (22.3%)	150 (77.7%)	
5-alpha-reductase deficiency	1 (9.1%)	10 (90.9%)	
Partial gonadal dysgenesis	1 (3.6%)	27 (96.4%)	
Mixed gonadal dysgenesis	2 (22.2%)	7 (77.8%)	
Hypogonadotropic hypogonadism	0 (0%)	10 (100%)	
17beta-hydroxysteroid dehydrogenase-3 deficiency	6 (50%)	6 (50%)	
Ovotesticular DSD	2 (40%)	3 (60%)	
Leydig cell hypoplasia	1 (7.1%)	13 (92.9%)	
Vanished testes	0 (0.0%)	9 (100.0%)	
21hydroxylase CAH	76 (84.4%)	14 (15.6%)	
3beta-hydroxysteroid dehydrogenase	2 (25.0%)	6 (75.0%)	
17α hydroxylase CAH	1 (33.3%)	2 (66.7%)	
POR CAH	0 (0.0%)	7 (100.0%)	
20,22 desmolase CAH	2 (100.0%)	0 (0.0%)	
Anatomical defects	7 (25.9%)	20 (74.1%)	
Isolated hypospadias	0 (0.0%)	9 (100.0%)	
Chromosomal aneuploidy	0 (0.0%)	2 (100.0%)	
• Prader score			P value
1	4 (100%)	0 (0%)	0.02
2	21(84.0%)	4 (16.0%)	
3	37 (92.5%)	3 (7.5%)	
4	27 (87.1%)	4 (12.9%)	
5	2 (40.0%)	3 (60.0%)	
• Quigley score (7)			<0.001
1	0 (0%)	28 (100%)	
2	0 (0%)	53 (100%)	
3	12 (7.4%)	151 (92.6%)	
4	20 (38.5%)	32 (61.5%)	
5	15 (48.4%)	16 (51.6%)	
6/7	24 (96.0%)	1 (4.0%)	
• EMS (12)			<0.001
0	1 (12.5%)	7 (87.5%)
1	2 (40%)	3 (60%)
2	26 (44.1%)	33 (55.9%)
3	35 (32.7%)	72 (67.3%)
4	1 (25%)	3 (75%)
5	4 (11.1%)	32 (88.9%)
6	2 (4.1%)	47 (95.9%)
7	0 (0%)	5 (100%)
8	0 (0%)	30 (100%)
9	0 (0%)	26 (92.9%)
10	0 (0%)	4 (100%)
11	0 (0%)	19 (100%)
• Karyotype			P value
46XX	83 (84.7%)	15 (15.3%)	<0.001
46XY	70 (20.8%)	266 (79.2%)
Others	3 (21.4%)	11 (78.6%)

CAIS, complete androgen insensitivity; PAIS, partial androgen insensitivity; EMS, external masculinization score; DSD, differences of sexual development; *p*<0.05 was considered significant.

**Table 3:** Comparison between patients with sufficient and inadequate HCG response regarding post stimulation hormonal levels.

HCG response		T-Post	DHT-Post	T/DHT	Δ4A-Post	DHEA-Post
		ng/ml	ng/dl		ng/ml	ng/ml
Poor	Mean	0.27	2.64		0.525	1.06
	Number	64	63		64	48
	SD	0.31	2.65		1.23	0.96
	Median	0.2	2		0.3	0.75
	Minimum	0.003	0.01		0.01	0.1
Good	Mean	3.42	22.32	13.9	0.77	1.1
	Number	211	211	211	211	156
	SD	2.54	19.44	8.35	1.32	0.99
	Median	2.7	17.3	13	0.3	0.8
	Minimum	0.2	0.6		0.04	0.1
p Value		<0.001	<0.001		0.18	0.84

Δ4A, Δ 4 androstendione; HCG, human chorionic gonadotropin; T, testosterone; DHT, dihydrotestosterone; DHEA, Dehydroepiandrosterone; *p*<0.05 is considered significant.

The mean ±SD of inhibin B level among 46,XY DSD patients was 174.38 pg/dl ±122.58. Fifty patients (38.8%) showed low levels of inhibin B to the age-appropriate reference ranges. The mean serum inhibin B level of patients with undescended testes (n = 58) was 204.74 pg/dl (range: 7–679) with a mean serum AMH level of 51.21 ng/ml (range: 0.01–490).

The median AMH in patients with poor HCG response was statistically significantly lower compared to the median AMH in patients with normal HCG response (*p*<0.001). However, there was no significant difference in serum inhibin B among patients with poor HCG response (*p*= 0.23) (**Table 4**). **Table 5** outlines the AMH and inhibin B state of patients diagnosed with different etiologies of 46,XY DSD.

**Table 4:** Comparison between patients with sufficient and inadequate HCG response with respect to AMH and Inhibin B levels.

HCG response		AMH level	Inhibin B
		ng/ml	pg/dl
Poor	Mean±SD	32.31 ± 41.05	160.88 ± 126
	Number	46	31
	Median	20.5	133.9
	Min–Max	0.01–187	13.5–650
Good	Mean±SD	82.69 ± 109.7	182.2 ± 129.55
	Number	34	76
	Median	59.15	151.2
	Min–Max	0.01–490	7–679
P Value		<0.001	0.23

AMH, antimullerian hormone; HCG, human chorionic gonadotropin; SD, standard deviation; Min, minimum; Max, maximum; *p*<0.05 was considered significant.

**Table 5:** AMH and inhibin B state among different etiologies of patients with 46,XY DSD.

Diagnosis	Inhibin B		*p* value	AMH		*P *value
	Normal	Low		Normal	Low	
CAIS	3 (100%)	0 (0%)	0.714			<0.001
PAIS	39 (60.9%)	25 (29.1%)		28 (90.3%)	3 (9.7%)	
5-alpha-reductase deficiency	1 (33.3%)	2 (66.7%)		1 (100%)	0	
Partial gonadal dysgenesis	8 (53.3%)	7(46.7%)		10 (52.6%)	9(47.4%)	
Hypogonadotropic hypogonadism	3 (50%)	3 (50%)		5(100%)	0 (0%)	
17beta-HSD deficiency	2 (33.3%)	4 (66.7%)		3 (100%)	0 (0%)	
Leydig cell hypoplasia	3 (75%)	1 (25%)		10 (90.9%)	1 (9.1%)	
Vanished testes	5 (83.3%)	1 (16.7%)		0 (0%)	2 (100%)	
17α hydroxylase CAH	1 (50%)	1 (50%)		1 (100%)	0 (0%)	
Anatomical defect	13 (76.9%)	3 (23.1%)		9 (90%)	1 (10%)	
20,22 desmolase CAH				1 (100%)	0 (0%)	

CAIS, complete androgen insensitivity; PAIS, partial androgen insensitivity; CAH, congenital adrenal hyperplasia; 17beta-HSD deficiency, 17beta-hydroxysteroid dehydrogenase-3 deficiency; AMH, antimullerian hormone; *p*<0.05 is considered significant.

**Figure 2:**
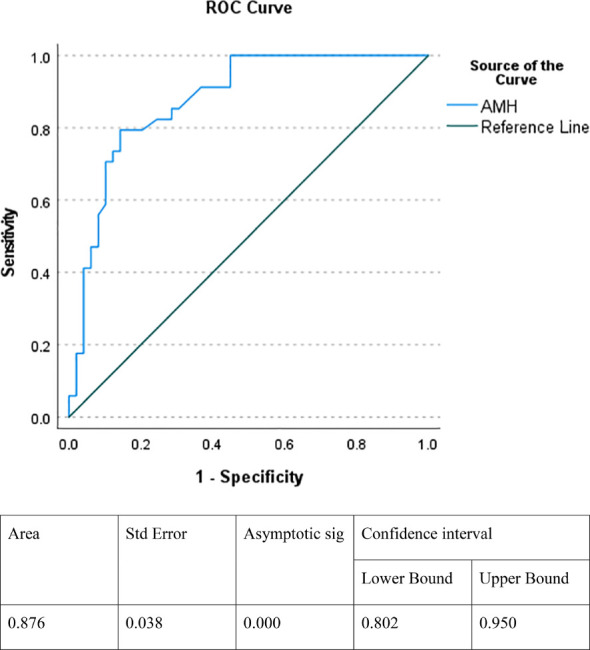
ROC curve indicating the specificity and sensitivity of AMH in detecting functioning testicular tissue. AMH, antimullerian hormone.

The cut off value of AMH, which indicates the presence of functioning testicular tissue, was 14.5 ng/ml (100% sensitivity and 55.1% specificity), and the Positive Predictive Value and Negative Predictive Value were 60.71% and 100.00%, respectively, using the ROC curve (**Figure 2**).

**Figure 3:**
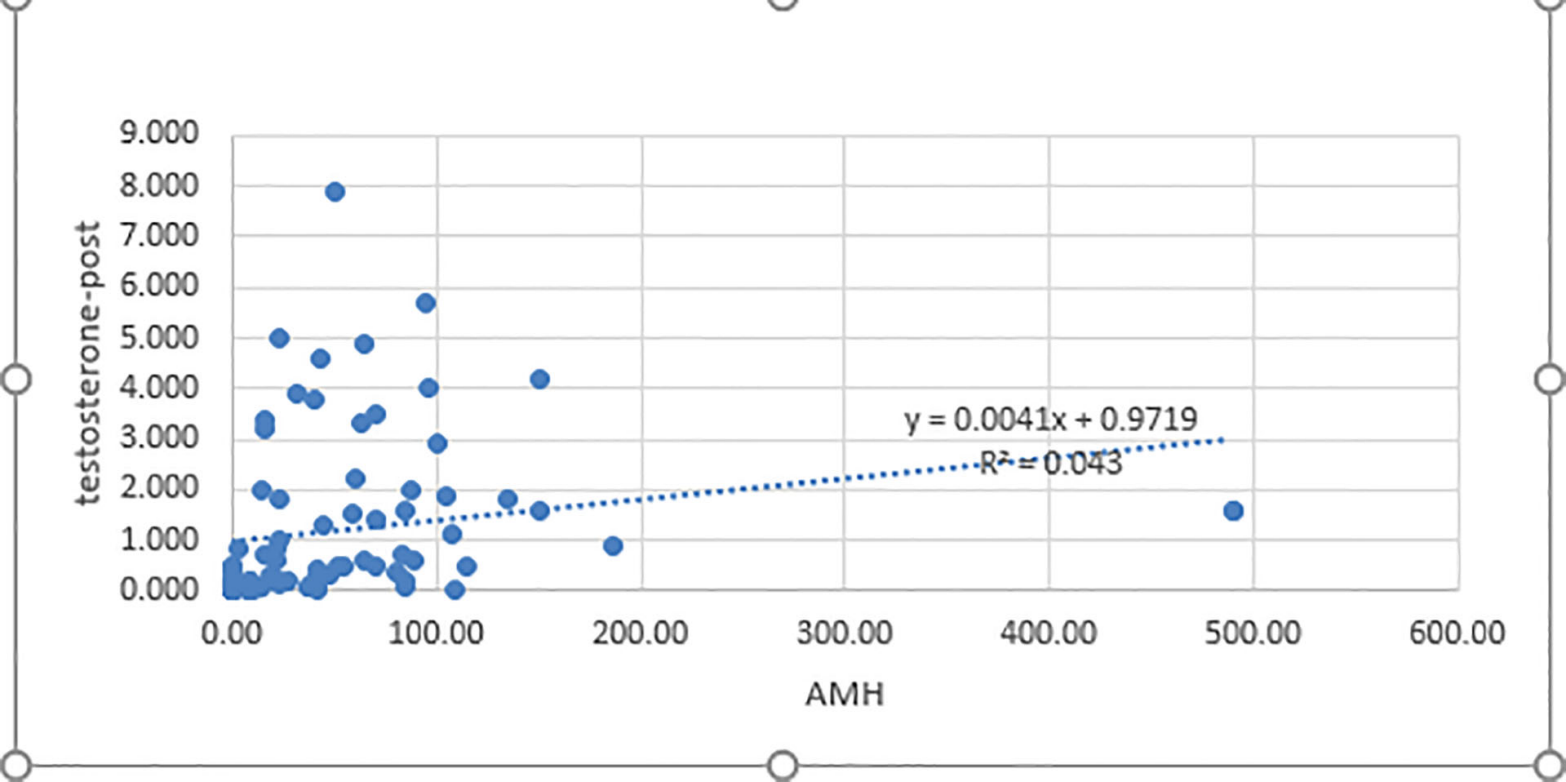
Correlation between AMH and post-HCG testosterone levels. AMH, antimullerian hormone; testosterone post: post-human chorionic gonadotropin test stimulation; *p<* 0.05 was considered significant.

Our results showed the AMH level was positively correlated with the post-HCG stimulation testosterone level (*p*<0.001, r= 0.42) and DHT level *(p*<0.001, r = 0.40) (**Figure 3**). In contrast, serum inhibin B was not significantly correlated with stimulated testosterone and DHT levels (*p* = 0.595 and *p* = 0.454, respectively).

## 4 Discussion

This study included 451 infants and children followed at the DEMPU, 76.7% of which presented with atypical genitalia, including isolated micropenis, isolated hypospadias, and isolated undescended testes. This finding was consistent with previous studies conducted in Pakistan and Turkey (20, 21). In addition to atypical genitalia, salt-losing manifestations were present in 21.3% of our patients. Patients who were diagnosed with complete androgen insensitivity (CAIS) presented either accidental palpable testes in labial folds or were accidently discovered during inguinal hernia, in accordance with the findings of Konar et al. (22). The mean age of presentation among patients without salt-losing manifestations was 3.5 years. This comes in contrast with studies performed in developed countries reporting neonatal presentation and early management, including surgical treatment, before the age of six months (23). A possible explanation for this delayed presentation in cases with atypical genitalia, which has also been reported by Nasir et al., may be that health care providers failed to perform a thorough examination of the genitalia (24). Also, contrary to the developed countries, ignorance of our conservative society with regard to DSD is a significant cause of delayed presentations of cases with atypical genitalia (25). However, patients with salt-losing manifestations, who represented 87.5% of all CAH patients, had earlier presentation during infancy and the neonatal period.

Patients with 46,XY DSD represented 74.5% of our patients while 21.7% had a 46,XX karyotype, which was consistent with the results of previous studies (4, 26). However, Al-Jurayyan et al., Dar et al. and Nasir et al. reported that 46,XX DSD were more prevalent than 46,XY DSD (24, 27, 28) All cases with the 46 XX karyotype were diagnosed as CAH except one patient who had SRY gene translocation. We included the CAH population in this study, but most women with CAH do not identify as having a DSD; they identify as having an adrenal steroid disorder (29).

PAIS was the most prevalent etiology of 46,XY DSD, representing 42.8% of all patients, a result that is consistent with the findings of Hafez et al. (9), as PAIS or 5-alpha-reductase deficiency represented 44.19% of their studied patients. The frequency of bilateral undescended testes was found to be 40.4% in our study, including patients with inguinal or nonpalpable testes. In contrast, the prevalence of this condition was 1.52% in a study conducted in Bulgaria on children aged <1 years up to 19 years old (30) and 20% in Pakistan (31).

There was a predilection for the male sex of rearing among under virilized males despite the high degrees of ambiguity; 51.6% and 61.5% of patients with Quigley scores 5 and 4, respectively, were assigned as males. Mazen et al. (4) reported similar findings in the Egyptian society.

Sertoli cells continue to release high levels of AMH during infancy and childhood (32). AMH has been considered a good biomarker for testicular function due to the little clinical use of basal testosterone and gonadotropin levels following minipuberty (33). Similarly, inhibin B is secreted by the sertoli cells in children and adults and can be used as a marker for testicular function in these populations (34).

Our results showed a positive correlation between AMH level and post-HCG stimulation DHT and testosterone levels. However, there was no significant correlation between the inhibin B level and stimulated DHT and testosterone levels. Hafez et al. found similar results regarding AMH; however, the authors found significant correlations between inhibin B and post-HCG stimulated testosterone and dihydrotestosterone (9). In addition, Kunini et al. found a significant correlation between inhibin B and post-HCG stimulated testosterone (35).

In our study, 90 % patients with PAIS, 100% of patients with 5-alpha-reductase deficiency and 90 % of those with anatomical defects had normal AMH levels, which was consistent with the results of Szarras-Czapnik et al. (36) Also Karaoglan reported normal AMH in patients with AIS and those with anatomical defects; however, the authors also found relatively low AMH levels in patients with 5-alpha-reductase deficiency (37). Our results demonstrated low AMH levels in more than half of patients with partial gonadal dysgenesis, which agreed with the results of Karaoglan (37).

## 5 Conclusions

In the present study, the most prevalent DSD category was patients with 46,XY DSD. However, CAH, predominantly classic salt-wasting CAH, was a major etiology of DSD. In patients with XY DSD, AMH was a reliable marker for detecting the presence and function of testes in infants and prepubertal children. Furthermore, AMH levels were a valuable tool for making differential diagnosis of DSD, with low AMH levels indicating global testicular disorders and normal or elevated AMH levels indicating disorders of androgen biosynthesis or action.

Our study has several limitations. First, this was a longitudinal study but there was no long-term follow-up. Second, some patients had missing data. Third, molecular analysis for specific disorders was not performed. However, our study has significant strengths as well. To our knowledge, this was the first study that evaluated the etiological diagnoses of DSD in the DEMPU, Faculty of medicine Cairo University. In addition, the large sample size included in our study allowed for a wide variety of etiological diagnoses, including rare conditions with detailed clinical profiles, with biochemical and histopathological confirmation whenever possible.

## Data availability statement

The original contributions presented in the study are included in the article/supplementary material. Further inquiries can be directed to the corresponding author.

## Ethics statement

The studies involving human participants were reviewed and approved by the scientific ethics committee of pediatric department, Faculty of Medicine, Cairo University on 12/10/2019 with code n: MD-92-2019. An informed consent was obtained from each child or his parents before enrollment. The consent was verbal, as our research was part of MD thesis of a candidate in our institute so it was performed under Cairo University regulations at the time of performance of the study. Written informed consent from the participants' legal guardian/next of kin was not required to participate in this study in accordance with the national legislation and the institutional requirements.

## Author contributions

SH conceived of the study, participated in its design, supervised data collections from the patients and helped to draft the manuscript. MM participated in study design and data interpretation coordination and helped to draft the manuscript. SA participated in the study design, data interpretation and coordination and helped to draft the manuscript. NR participated in the study design, supervised collecting data and laboratory work and helped to draft the manuscript. EA participated in patient selection and data collection. All authors contributed to the article and approved the submitted version.
